# Antimicrobial promotion of pig growth is associated with tissue-specific remodeling of bile acid signature and signaling

**DOI:** 10.1038/s41598-018-32107-9

**Published:** 2018-09-12

**Authors:** Ignacio R. Ipharraguerre, Jose J. Pastor, Aleix Gavaldà-Navarro, Francesc Villarroya, Alessandro Mereu

**Affiliations:** 1 Institute of Human Nutrition and Food Science, University of Kiel, Hermann-Rodewald-Strasse 6-8, D-24128 Kiel, Germany; 2 Innovation Division, Lucta S.A., Parc de Recerca UAB, Edifici Eureka, 08193 Bellaterra, Catalonia Spain; 3 Departament de Bioquímica i Biomedicina Molecular, Institut de Biomedicina (IBUB), Universitat de Barcelona, and CIBER Fisiopatología de la Obesidad y Nutrición, Avinguda Diagonal 645, Edifici nou Pl.-1, 08028 Barcelona, Catalonia Spain; 4 Present Address: Yara Iberian, C/ Infanta Mercedes 31 – 2nd floor, 28020 Madrid, Spain

## Abstract

The spread of bacterial resistance to antimicrobials (AMA) have intensified efforts to discontinue the non-therapeutic use of AMA in animal production. Finding alternatives to AMA, however, is currently encumbered by the obscure mechanism that underlies their growth-promoting action. In this report, we demonstrate that combinations of antibiotics and zinc oxide at doses commonly used for stimulating growth or preventing post-weaning enteritis in pigs converge in promoting microbial production of bile acids (BA) in the intestine. This leads to tissue-specific modifications in the proportion of BA, thereby amplifying BA signaling in intestine, liver, and white adipose tissue (WAT). Activation of BA-regulated pathways ultimately reinforces the intestinal protection against bacterial infection and pathological secretion of fluids and electrolytes, attenuates inflammation in colon and WAT, alters protein and lipid metabolism in liver, and increases the circulating levels of the hormone FGF19. Conceivably, these alterations could spare nutrients for growth and improve the metabolic efficiency of AMA-treated animals. This work provides evidence that BA act as signaling molecules that mediate host physiological, metabolic, and immune responses to the AMA-induced alterations in gut microbial metabolism, eventually permitting the growth-promoting action of AMA. Consequently, BA emerge as a promising target for developing efficacious alternatives to AMA.

## Introduction

For more than 50 years, low doses of antibiotics either alone or in combination with pharmacological levels of heavy metals (i.e., zinc and copper) have been widely used in swine production to accelerate growth^1,2^. More recently, oral administration of similar levels of these antimicrobial agents (AMA) but for disease prevention has become recurrent in pig farming, particularly in countries where antibiotic growth promoters have been banned^3,4^. An expanding body of evidence, however, links such practices to the spread of antibiotic-resistant bacteria in animals and humans^5,6^. It is crucial therefore to find ways for reducing the overuse of AMA in food-producing animals without jeopardizing their health and wellbeing as well as the economic viability of farm operations. Intriguingly, AMA consistently promote weight gain irrespective of their bacterial spectrum^1^, but a uniting mechanism underlying their growth-enhancing action remains elusive. As expected, this uncertainty hinders the development of alternative strategies to AMA^7,8^.

A number of studies have shown that AMA-induced weight gain is accompanied by alterations in gut microbial ecology, which frequently entails suppression of gram-positive commensals and proliferation of bacterial communities with enhanced capacity for dietary energy harvest^9–12^. These changes include increased lipid absorption by the host owing to reduced activity of bacterial bile salt hydrolases (BSH) and the associated deconjugation of bile salts in the intestine^13^. In addition, several data sets indicate that the growth-permitting effect of AMA also involves reduced inflammation and immune activation (mainly in the intestinal mucosa), although the underlying mechanism is currently unclear and evidence from animals other than mice is scant^14–19^. In addition to these effects, emerging data from murine models suggest that AMA can cause variations in the biosynthesis of bile acids (BA) and the instructive functions that they exert in the metabolic and immune interplay between the host and its gut microbiota. Such functions are primarily mediated by the nuclear receptor farnesoid X receptor (FXR) and the G protein-coupled receptor TGR5 (TRG5), both of which are involved in the regulation of BA, energy, and immune homeostasis in the host^20,21^. In this regard, disruption of mouse microbiota by antibiotics not only altered synthesis and enterohepatic cycling of primary (produced by the liver) and secondary (produced by bacteria) BA but also affected BA signature and FXR signaling in a tissue-specific manner^22,23^. Furthermore, both increased BSH activity due to expression of cloned BSH in the gut^24^ and decreased BSH activity due to suppression of intestinal *Lactobacilli*^25^ were associated with weight gain in rodents. The explanation for this apparent contradiction involves differential patterns of BA signaling in the intestine and liver in response to opposite fluctuations in the local concentration of FXR-antagonist primary bile salts and FXR-agonist secondary BA^24,25^. An additional feature of BA is their toxicity to bacteria, which contributes to maintain microbial balance within the gut^26^. Dysregulation of bacterial transformations in the large bowel, in particular 7α-dihydroxylation, was recently found to correlate with dysbiosis and concomitant intestinal pathologies^27^. By interacting with intestinal FXR and TGR5, BA were also shown to induce mechanisms that protect the gut epithelium against barrier dysfunction, bacteria invasion, and overstimulation of the immune-inflammatory axis^28,29^.

Collectively, evidence supports the hypothesis that alterations in BA metabolism, signature, and signaling represent a conceivable mechanism for the growth-promoting action of the non-therapeutic use of AMA. Certainly, this mechanism integrates many of the previously proposed effects of AMA^11,13–15,30,31^. It is important to note, however, that the chemical structure of BA, including conjugation and degree of hydroxylation, determines their ability to activate or inhibit signaling pathways and major differences in BA chemistry exist across animal species^32^. Moreover, the regulatory functions of BA in food-producing animals and the influence that customary combinations of AMA rather than single interventions have on them remain largely unknown. In this report, data from two independent studies are presented. The first study investigated the impact of a combination of zinc oxide, amoxicillin, and colistin (ZAC) (Supplementary Table S1) on BA metabolism, profile and regulatory actions in the intestine and liver of weaned piglets. Two years later, a second study was conducted to confirm and expand findings from the first study. To this end, a combination of zinc oxide, chlortetracycline, and tiamulin (ZCT) (Supplementary Table S2) was used to cover a different bacterial spectrum than ZAC and BA instructive functions were mainly examined in tissues outside the enterohepatic system. For these reasons, not all biomarkers were measured in both studies. We chose ZAC and ZCT because both AMA combinations are extensively used in commercial pig production in Europe to prevent weaning-induced enteric disorders and growth check. This work identifies BA as integrators and modulators of the host physiological, metabolic, and immune-inflammatory responses to the AMA-mediated alterations in gut microbial metabolism, which makes them a promising target for the development of efficacious alternatives to AMA.

## Results

### Antimicrobials promote growth independently of the metabolic phenotype

**Figure 1:**
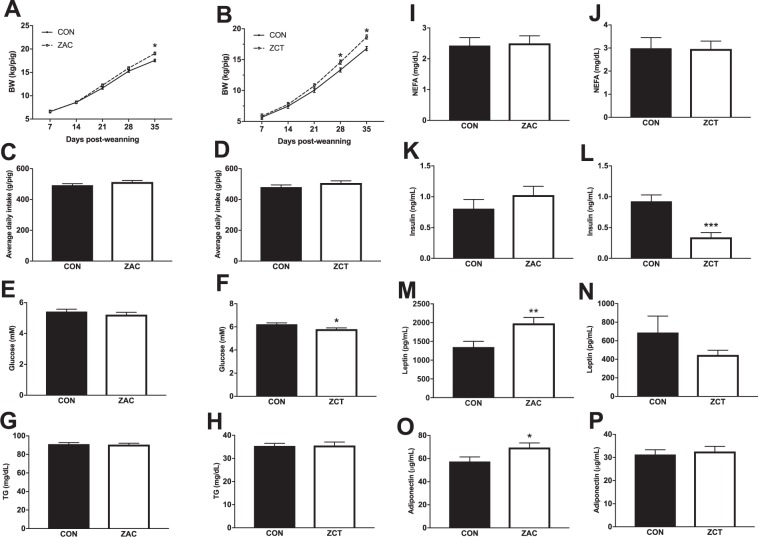
Growth promotion by in-feed antimicrobials (AMA) is paralleled by inconsistent alterations in the metabolic profile of pigs. Two independent cohorts of piglets were weaned and fed for 35 days cereal-based diets medicated with combinations of AMA, which were either zinc oxide, amoxicillin, and colistin (ZAC) or zinc oxide, chlortetracycline, and tiamulin (ZCT) at dosing levels routinely used for preventing bacterial enteritis and stimulating pig growth. Control pigs (CON) received no AMA. Plasma collected on days 34 and 35 were used to measure biomarkers by ELISA. Data from the two experiments were combined in the figure. (**A**,**B**) Weight gain, (**C**,**D**) average daily feed intake, and circulating concentration of metabolites (**E**–**J**) and hormones (**K**–**P**) in pigs fed CON or medicated (ZAC and ZCT) diets. Data were analyzed with ANOVA. Least squares means ± SEM are plotted, ^*^P < 0.05, ^**^P < 0.01, *n* = 6–12.

Following practices currently used in commercial pig production, two cohorts of piglets from the same commercial operation were weaned averaging 23 ± 2 days of age, transported to a research nursing facility, and therein fed for 35 days cereal-based diets medicated with widely used combinations of AMA in two independent experiments. Antimicrobial combinations were ZAC or ZCT at dosing levels routinely used for preventing bacterial enteritis and stimulating pig growth. Control piglets (CON) received no AMA. In both experiments, AMA-fed pigs gained more body weight (BW) than their control counterparts. The magnitude of this effect was similar for the two AMA combinations, which totaled at the end of the experiment 1.6 (9%) and 1.9 (11%) kg of BW for pigs fed ZAC or ZCT, respectively (Fig. 1A and B). Consistent with previous findings^1^, such responses were not associated with differences in feed intake between CON and ZAC (Fig. 1C) or CON and ZCT (Fig. 1D). A possible mechanistic component of the growth-permitting action of AMA is enhanced energy absorption, particularly as lipids^13^. As compared with CON, however, ZAC increased the circulating levels of leptin (Fig. 1M) and adiponectin (Fig. 1O), whereas ZCT reduced the plasma concentrations of insulin (Fig. 1L) and glucose (Fig. 1F), and none of them affected lipid fractions in circulation (Fig. 1G–J).

### Antimicrobials converge in altering bile acid biosynthesis by gut microbiota

**Figure 2:**
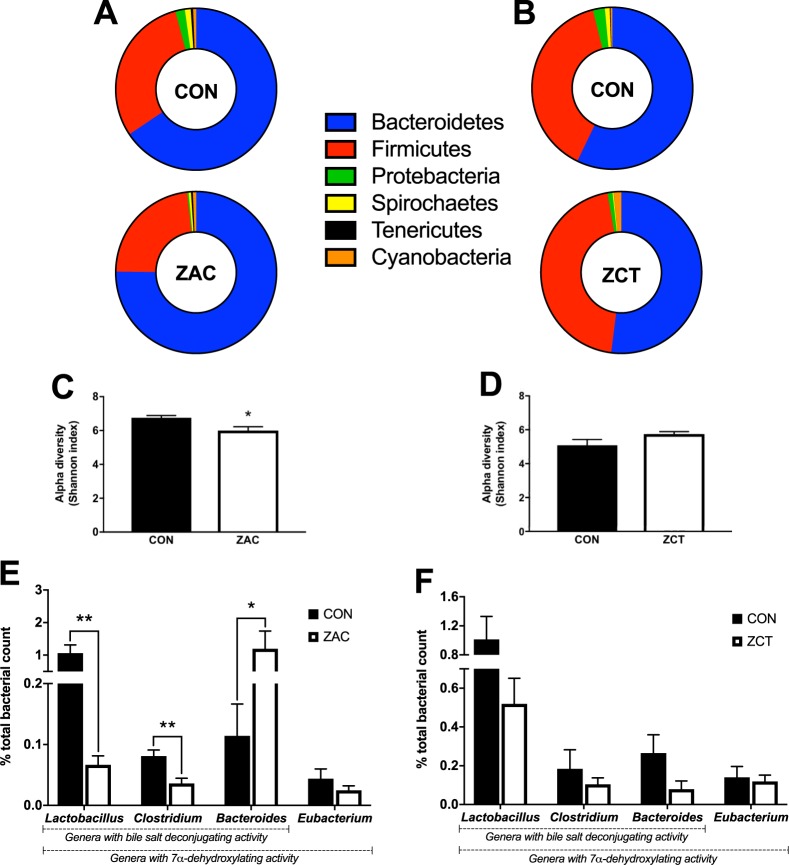
In-feed antimicrobials (AMA) have irregular influence on the intestinal microbiota of pigs. Two independent cohorts of piglets were weaned and fed for 35 days cereal-based diets medicated with combinations of AMA, which were either zinc oxide, amoxicillin, and colistin (ZAC) or zinc oxide, chlortetracycline, and tiamulin (ZCT) at dosing levels routinely used for preventing bacterial enteritis and stimulating pig growth. Control pigs (CON) received no AMA. Colonic contents obtained on days 34 and 35 were used to assess the microbiome profile by massive sequencing of the hypervariable regions V1-V2 of the 16 S rRNA gene. Data from the two experiments were combined in the figure. (**A**,**B**) Composition (percent reads at the phylum level) and (**C**,**D**) alpha diversity of colonic microbiota from pigs fed CON or medicated (ZAC and ZCT) diets. (**E**,**F**) Proportion of bile-acid metabolizing bacteria (percent reads at the genus level) in colonic contents of pigs fed ZAC (**E**) or ZCT (**F**). Data were analyzed with Student t test (percent reads) and non-parametric Montecarlo permutation test (alpha diversity). Means ± SEM are plotted, ^*^P < 0.05, ^**^P < 0.01, *n* = 10–12.

**Figure 3:**
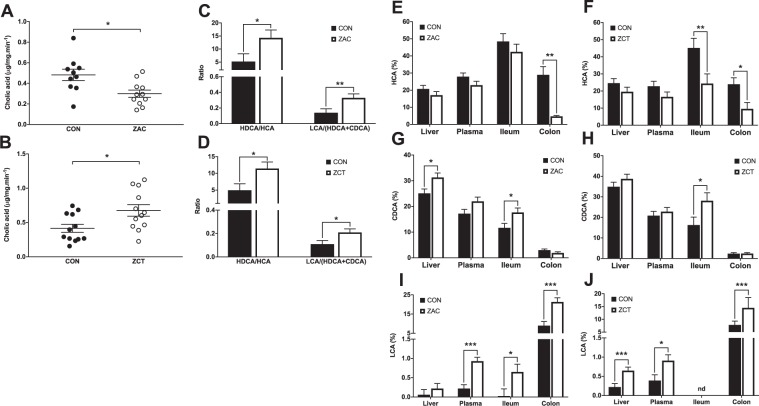
Feeding antimicrobials (AMA) to pigs alter the synthesis of secondary bile acids and the profile of bile acids (BA) in a tissue-specific manner. Two independent cohorts of piglets were weaned and fed for 35 days cereal-based diets medicated with combinations of AMA, which were either zinc oxide, amoxicillin, and colistin (ZAC) or zinc oxide, chlortetracycline, and tiamulin (ZCT) at dosing levels routinely used for preventing bacterial enteritis and stimulating pig growth. Control pigs (CON) received no AMA. The concentration of BA was measured via UPLC-MS in samples taken on days 34 and 35. Data from the two experiments were combined in the figure. (**A**,**B**) Bile salt hydrolase activity (assessed by treating colonic digesta with labeled tauro-cholic-d5 acid and measuring the formation of cholic acid-d5 via UPLC-MS) and (**C**,**D**) primary to secondary BA ratios in colonic contents of pigs fed CON or medicated (ZAC and ZCT) diets. (**E***–***J**) Relative concentration of HCA **(E**,**F**), CDCA (**G**,**H**), and LCA (**I**,**J**) in liver, plasma, and intestinal mucosa of pigs fed ZAC (**E**,**G** and **I**) or ZCT (**F**,**H** and **J**). Data were analyzed with ANOVA. Least squares means ± SEM are plotted, ^*^P < 0.05, ^**^P < 0.01, *n* = 10–12, nd = not detected.

To assess the impact of AMA on gut microbiota, samples of colonic contents from both cohorts were analyzed by massive sequencing of the V1-V2 regions of the 16 S rRNA gene. At the phylum level, all groups presented microbial compositions (Fig. 2A and B) representative of the metagenome prevailing in healthy pigs of similar age^10^. Compared with CON, however, ZAC favored the proliferation of *Bacteroidetes* (65 vs. 74%) mainly at the expense of *Firmicutes* (30 vs. 23%) and *Proteobacteria* (1.9 vs. 0.3%) resulting in reduced microbial diversity within (Fig. 2C) and between individuals (Supplementary Table S3). The major BA biotransformations that take place in the intestine of humans and rodents are carried out by bacteria that possess bile salt hydrolase (BSH) activity and/or 7α-dihydroxylating capacity^33^. Concerning these bacteria, ZAC drastically decreased the proportion of *Lactobacillus* and *Clostridium* whereas increased the relative abundance of *Bacteroides* (Fig. 2E). In contrast, the colonic microbiota of ZCT-fed pigs (Fig. 2B and D and Supplementary Table S3), including the proportion of BA-biotransforming bacteria (Fig. 2F), did not differ substantially from that of their CON counterparts. Furthermore, whereas ZAC depressed the BSH activity of colonic contents, ZCT resulted in the opposite effect (Fig. 3A and B).

Despite their differential effects on gut microbial ecology and BSH activity, both AMA combinations robustly enhanced secondary to primary BA ratios in colon (Fig. 3C and D), indicating that AMA administration stimulated microbial conversion (7α-dehydroxylation) of hyocholic acid (HCA) into hyodeoxycholic acid (HDCA) and of HDCA and chenodeoxycholic acid (CDCA) into lithocholic acid (LCA). Even though the microbial identity remains unclear, these findings demonstrate that combinations of AMA that are traditionally used in pig production for growth promotion or disease prevention coincide in altering in a similar way the metabolism of BA by gut microbiota irrespective of their bacterial spectrum.

### Antimicrobials modify the profile of bile acid in multiple tissues

Given the converging influence of AMA combinations on bacterial BA biosynthesis, we then measured BA in multiple compartments of the enterohepatic system. The ileal epithelia of CON and medicated pigs were enriched with BA before bile salts (Supplementary Fig. S1). The concentrations of total BA in colon and liver, but not in ileum and plasma, were reduced by ZCT (Supplementary Fig. S2). The profile of BA, however, was markedly altered by both AMA combinations in all examined tissues. Pigs treated with ZAC and ZCT shared a reduction in the proportion of HCA in colonic mucosa accompanied by an increase in the percentage of CDCA in ileal epithelium plus a remarkable surge in the ratio of LCA both in colonic mucosa and systemic circulation (Fig. 3E–J). In addition to these common alterations, ZAC produced an increase in hepatic CDCA (Fig. 3G) and ileal LCA (Fig. 3I), whereas ZCT caused a decline in ileal HCA (Fig. 3F) along with an increase in hepatic LCA (Fig. 3J). Because LCA and CDCA are the most potent endogenous agonists of BA receptors^20,34^, these results denote that administering AMA to pigs at levels shown to stimulate growth coincide in expanding the potency of the BA pool within and beyond the enterohepatic system.

### Antimicrobials enhance the circulating concentration of FGF19

**Figure 4:**
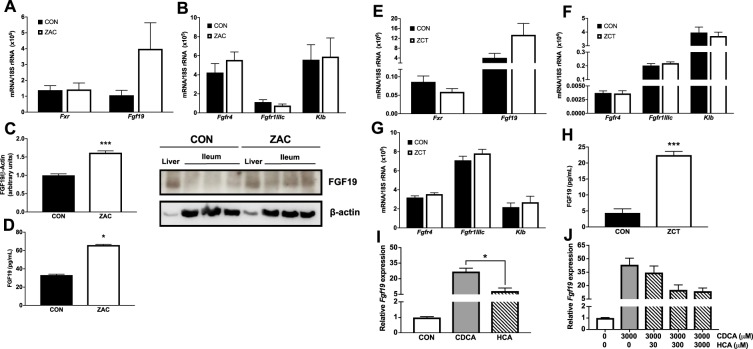
Feeding antimicrobials to pigs enhance the circulating concentration of FGF19 by targeting intestinal FXR. Two independent cohorts of piglets were weaned and fed for 35 days cereal-based diets medicated with combinations of AMA, which were either zinc oxide, amoxicillin, and colistin (ZAC) or zinc oxide, chlortetracycline, and tiamulin (ZCT) at dosing levels routinely used for preventing bacterial enteritis and stimulating pig growth. Control pigs (CON) received no AMA. Gene expression was measured by qRT-PCR and protein levels by Western blot and ELISA in tissue samples taken on days 34 and 35 or ileal explants. Data from the two experiments were combined in the figure. (**A**,**B**) Ileal expression of genes encoding components of the FGF19 system and (**C**,**D**) concentration of FGF19 protein in ileum (**C**) and plasma (**D**) of pigs fed CON or ZAC. (**E–G**) Expression of genes encoding components of the FGF19 system in ileum (**E**), sWAT (**F**), and skeletal muscle (**G,H**) plasma concentration of FGF19 protein in pigs fed CON or ZCT. In C, FGF19 and β-actin specific bands are displayed from cropped blots. Full-length blots are presented in Supplementary Figure S7. Data were analyzed with ANOVA. Least squares means ± SEM are plotted, ^***^P < 0.001, *n* = 9–12 per group. (**I,J**) Expression of *Fgf19* in ileal explants treated with 300 μM of CDCA or HCA (**I**) or with 3000 μM of CDCA in the presence of different amounts of HCA (**J**). Data were analyzed with Student t test. Means ± SE are plotted, ^*^P < 0.05. This assay was performed using explants from 4 independent pigs from the CON group.

To elucidate the implications of the AMA-induced alterations in intestinal BA signature we next examined the BA-FXR-FGF19 signaling pathway in ileum and potential target tissues of this enterokine. We observed that *Fxr*, *Fgf19*, and porcine genes encoding FGF19 receptors (*Fgfr4*, *Fgfr1IIIc*, and *Klb*) were expressed in ileal mucosa (Fig. 4A,B and E). Consistent with data from rodents^35–37^, we also found detectable levels of FGF19 receptors, including *Klb*, in liver (Supplementary Fig. S3), WAT (Fig. 4F and Supplementary Fig. S3) and muscle (Fig. 4G). Not significant differences between CON and AMA groups were detected in the mRNA level of ileal *Fgf19* (Fig. 4A) and its receptors across examined tissues (Fig. 4B and E–G). Compared to CON, however, ZAC-fed pigs had higher expression of FGF19 protein in ileum (Fig. 4C) and animals from both AMA groups experienced a substantial increase (≥ 1-fold) in the circulating concentration of FGF19 (Fig. 4D and H). These results indicate that in pigs AMA coincide in enhancing BA signaling through the intestinal FXR-FGF19 pathway. They also reveal that, like in rodents^35–37^, key tissues involved in the metabolic regulation of growth possess the signaling machinary required for responding to FGF19.

These findings suggest that in pigs CDCA is a more potent FXR agonist than HCA. To explore this possibility, we treated ileal explants from CON pigs with these BA and found that, at the same concentration, CDCA resulted in a 2-fold higher expression of *Fgf19* than HCA (Fig. 4I). Furthermore, the addition of incremental concentrations of HCA into the culture media failed to augment the CDCA-induced expression of *Fgf19*, which actually followed a diminishing trend (Fig. 4J). Taken together, data indicate that the capacity of CDCA to signal through the FXR-FGF19 axis is higher than that of HCA.

### Intestinal protection by antimicrobials is associated with activation of FXR and TGR5 in the intestine

**Figure 5:**
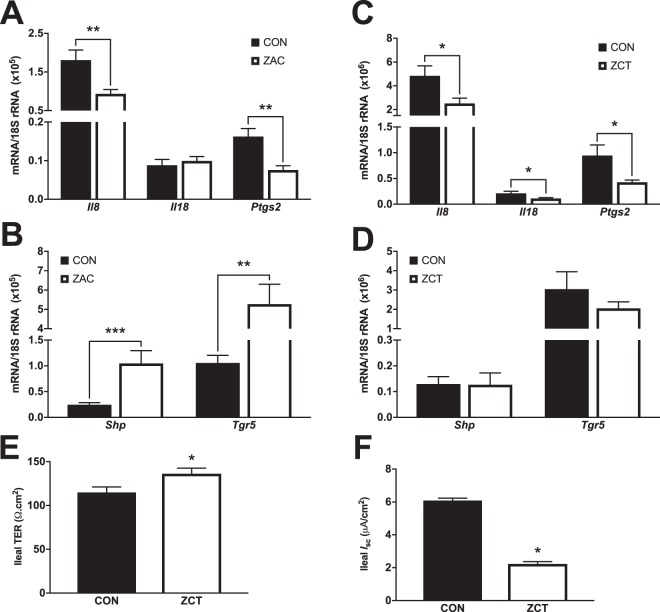
The enteroprotective action of in-feed antimicrobials in pigs is associated with increased signaling through intestinal FXR and TGR5. Two independent cohorts of piglets were weaned and fed for 35 days cereal-based diets medicated with combinations of AMA, which were either zinc oxide, amoxicillin, and colistin (ZAC) or zinc oxide, chlortetracycline, and tiamulin (ZCT) at dosing levels routinely used for preventing bacterial enteritis and stimulating pig growth. Control pigs (CON) received no AMA. Gene expression was measured by qRT-PCR in intestinal mucosa samples taken on days 34 and 35. Data from the two experiments were combined in the figure. (**A**,**C**) Expression of proinflammatory genes in colon and (**B**,**D**) FXR-target genes in ileum of pigs fed CON or medicated (ZAC and ZCT) diets. Data were analyzed with ANOVA. Least squares means ± SEM are plotted, ^*^P < 0.05, ^**^P < 0.01, ^***^P < 0.001, *n* = 8–12 per group. (**E**) Transepithelial electrical resistance (TER) and (**F**) ion transport (*I*_sc_) in the ileal mucosa of pigs fed CON or ZCT. Data were analyzed with ANOVA. Least squares means ± SEM are plotted, ^*^P < 0.05, *n* = 8. This assay was performed by mounting mucosal segments from the mid-ileum in Ussing chambers from CON and ZCT-treated pigs.

At doses tested herein, AMA have proven ability to prevent intestinal dysfunction and inflammation in mice^14,15^ and farm animals^2,31,38,39^. Coincidentally, the BA sensors FXR and TGR5 participate in the regulation of numerous mechanisms implicated in the defense of the intestine^28,29,40^, suggesting that altered BA profile and signaling may be mechanistic components of the AMA enteroprotective action. We found that, compared with CON, the colon of ZAC-treated pigs had modified expression of immune genes regulated by BA sensors^28,40^, including reduced mRNA levels of the inflammatory genes *Il8* and *Ptgs2* (Fig. 5A) and increased transcripts of *Ang1* (Supplementary Fig. S4), which encodes a protein (agiogenin 1) with antibacterial and antimycotic effects^28^. These changes were accompanied by augmented BA signaling in colonic mucosa of ZAC animals, as denoted by the upregulated expression of genes whose transcription is controlled by FXR (Fig. 5B) or TGR5 (Supplementary Fig. S4). Administration of ZCT also repressed the expression of proinflammatory genes in colon (Fig. 5C) but did not affect other genes responsive to BA (Fig. 5D and Supplementary Fig. S4).

Early weaning, as carried out in our experiments, disrupts intestinal barrier function in pigs^41^ and activation of FXR^42^ or TGR5^29^ by exogenous ligands prevents the development of this pathology in murine models of intestinal inflammation. In assays with Ussing chambers, we observed that the ileal mucosa of ZCT-treated pigs had increased transepithelial electrical resistance (Fig. 5E), indicating improved function of the intestinal barrier compared with CON animals. Furthermore, ZCT reduced ileal ion transport (Fig. 5F) which is affected in the opposite direction by weaning^41^ as result of excessive secretory activity of the intestine (diarrhea). Collectively, these findings confirm the enteroprotective function of AMA in pigs and support the proposition that their mode of action involves reinforced BA signaling via intestinal BA sensors.

### Antimicrobials remodel bile acid signaling in liver and adipose tissue but not in muscle

**Figure 6:**
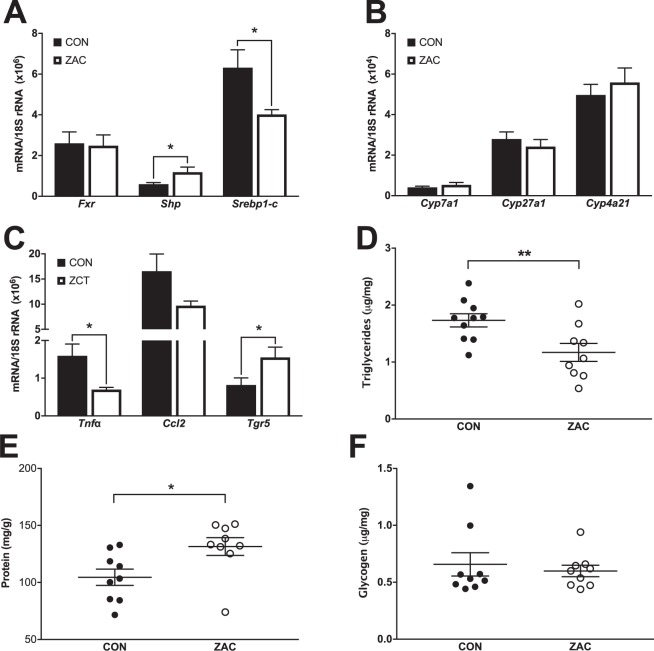
Feeding antimicrobials to pigs alter bile acid signaling in liver and subcutaneous white adipose (sWAT). Two independent cohorts of piglets were weaned and fed for 35 days cereal-based diets medicated with combinations of AMA, which were either zinc oxide, amoxicillin, and colistin (ZAC) or zinc oxide, chlortetracycline, and tiamulin (ZCT) at dosing levels routinely used for preventing bacterial enteritis and stimulating pig growth. Control pigs (CON) received no AMA. Samples taken on days 34 and 35 were used to measure gene expression in liver and sWAT by qRT-PCR and metabolites in liver by ELISA. Data from the two experiments were combined in the figure. (**A**,**B**) Hepatic expression of genes involved in the regulation of bile acid and lipid homeostasis and (**D–F**) concentration of triglycerides (**D**), protein (**E**), and glycogen (**F**) in liver of pigs fed CON or ZAC. (**C**) Expression of TGR5 and proinflammatory genes in sWAT of pigs fed CON or ZCT. Data were analyzed with ANOVA. Least squares means ± SEM are plotted, ^*^P < 0.05, ^**^P < 0.01, *n* = 9–12 per group.

After confirming that AMA combinations affected BA metabolism and signaling in the intestine, we then focused on tissues serving key roles in the metabolic regulation of growth. Compared with CON, administration of ZAC to pigs promoted activation of hepatic FXR as indicated by the counterregulatory expression (Fig. 6A) of *Shp*, a transcriptional corepressor induced by FXR, and *Srebp1-c*, a transcriptional activator of hepatic lipogenesis repressed by FXR via SHP^20^. In line with these findings, ZAC-treated pigs had lower concentration of triglycerides (TAG) in the liver than their CON counterparts (Fig. 6D). They also presented a higher content of hepatic protein (Fig. 6E), which is in agreement with the regulatory function that FGF19 plays in this organ^43^. Interestingly, transcript levels of enzymes implicated in BA synthesis were not affected by ZAC (Fig. 6B).

In mouse, BA also target adipose tissue, where upon activation of TGR5 induce energy expenditure via increased expression of *Dio2* in the context of diet-induced obesity^20^. As compared with CON animals, the subcutaneous WAT (sWAT) of ZCT-treated pigs presented increased expression of *Tgr5* (Fig. 6C) but not of *Dio2* and genes involved in thermogenesis and fatty acid oxidation (Supplementary Fig. S5). Instead, administration of ZCT reduced the mRNA abundance of *Tnfα* in sWAT (Fig. 6C), a proinflammatory cytokine downregulated by BA through a TGR5-dependent mechanism^44^. Even though *Tgr5* was expressed at detectable levels in skeletal muscle, none of the AMA-induced effects on liver and sWAT were observed in this tissue (Supplementary Fig. S6).

## Discussion

Clarifying the mechanisms by which AMA stimulate weight gain in farm animals is an endeavor of vital importance that requires disentangling the impact of AMA on the constantly evolving crosstalk between the host and its gut microbiota. The complexity entailed by such interactions has encouraged the use of reductionist models based on animals (mice), interventions (single medications), and/or conditions (highly-controlled environments and diets) that, albeit valuable, are distant from the customary use of AMA in animal production and the features (e.g., animal species, husbandry practices, hygiene, etc.) that characterize it^11,14–16^. Using an experimental approach that closely resembles intensive systems of pig production, we show that combinations of AMA with different bacterial spectrum at doses commonly used for stimulating growth and preventing post-weaning enteritis converge in promoting BW gain and altering the metabolism of BA by gut microbiota as well as the profile and signaling of BA in intestinal mucosa, liver, and WAT. In turn, these effects induce growth-supporting adaptations in pathways regulated by BA that are implicated in the immunometabolic regulation of BW. Such alterations include improved immune tolerance and barrier function of the intestinal mucosa, reduced inflammation in colon and WAT, and, most intriguingly, enhanced circulating levels of FGF19. This work provides evidence that BA serve a mechanistic role in the growth-permitting action of AMA by acting as signaling molecules that mediate host physiological, metabolic, and immune-inflammatory responses to the AMA-induced alterations in gut microbial metabolism.

In recent years, it has become clear that BA are regulatory molecules with endocrine functions that signal changes in the immunometabolic interplay between the host and its gut microbiota^34^. Several studies have shown that upsetting the intestinal microbiome of rodents with therapeutic doses of antibiotics causes profound alterations in BA profile, tissue distribution, and expression of genes that are involved in BA, energy, and immune homeotasis^22,45–47^. Interestingly, at sub-therapeutic levels the oral administration of antibiotics to mice resulted in less consistent shifts in microbial composition; yet, supported host growth, altered lipid metabolism, and attenuated the immune response of the intestinal mucosa^11,14,15^ through pathways that could possibly be regulated by BA^20,28,29,40^. Mirroring outcomes from murine models, the non-therapeutic addition of antibiotics into the diet of pigs had irregular influence on intestinal microbes^18^ but consistently enhanced BW gain and reduced the circulating concentration of biomarkers of inflammation^17^ and immune activation^18^. In addition to antibiotics, the feeding of high levels (≥2400 ppm) of zinc oxide to young pigs, a widely used AMA in swine production, stimulated growth with concurrent generation of gut microbial patterns and intestinal immune phenotypes resembling the ones generated by antibiotic growth promoters in mice^9,12,48^. Collectively, available data suggest a causal link between the gut microbiota-BA axis and the growth-enhancing action of AMA with diverse bacterial spectrum (define herein as low doses of antibiotics and/or high doses of zinc oxide). Nevertheless, the implication of BA in mediating physiological, metabolic, and immune responses to AMA has not been established. Furthermore, little is known about the regulatory functions of BA in food-producing animals, including pigs.

We confirmed in two independent studies that irrespective of their chemical strucuture and  bacterial spectrum both AMA interventions consistently increased weight gain by 9 (ZAC) and 11% (ZTC), agreeing with previous results and the notion that the antimicrobial specificity of AMA do not determine their growth-enhancing action^1^. Evidence from studies with mice^11,14,15^ and pigs^12,18,49,50^ clearly indicates that the mode of action of AMA involves changes in the gut microbiome. Such alterations could entail selection of bacterial species and genes with superior capacity for producing volatile fatty acids and thereby enhance the extraction of dietary energy by the host^11,50^. Additionally, this effect may be amplified by the suppression of bacteria bearing BSH activity, in particular *Lactobacillus* species, which is predicted to augment the concentration of bile salts in the intestinal lumen and, as a consequence, the emulsification and absorption of lipids^13^. However, a consistent taxonomical pattern or microbial enterotype is certainly not recurrent across published data, raising questions about the universality of this mechanism. In line with this observation, we found that ZAC and ZCT had inconsistent effects on the diversity and taxonomy of colonic microbes (ZAC influenced them whereas ZCT did not), the abundance of BSH-containing bacteria (ZAC repressed them but ZCT had no effects), and the activity of BSH in intestinal contents (ZAC decreased it whereas ZCT did the opposite). In part, these findings agree with previously established (positive) associations between antibiotic-induced BW gain and proliferation of *Lactobacillus*^49^ and between BW loss and both enhanced^24^ and reduced^25^ BSH activity in the gut. Furthermore, ZCT reduced circulating glucose and insulin while ZAC increased plasma levels of adiponectin and leptin. These responses are in conflict with the metabolic profile associated with high-energy-yielding microbiota^11^ and suggest that AMA actually improved energy utilization (insulin sensitivity)^51^ rather than energy supply. It is therefore apparent that an energy-yielding rearrangement of the structure or metabolism of gut microbiota is not uniformly related to the growth-permitting action of AMA.

Based on the expanding body of evidence that links diet-induced modifications of BA metabolism by intestinal microbes to metabolic and health disorders in humans and rodents^34,52^, we next investigated the impact of AMA on the microbial biosynthesis of BA and showed that ZAC and ZCT coincided in increasing the formation of HDCA and LCA in colon. This finding reveals that both AMA combinations enhanced bacterial removal of the 7α-hydroxy group in HCA and CDCA (7α-dehydroxylation), which quantitatively is the predominant microbial BA transformation in the gut^53^. We also demonstrated that ZAC and ZCT had a congruent impact on the BA signature in immunometabolically relevant tissues, mainly characterized by a widespread enrichment in LCA. Expectably, the most affected tissue was the intestine, where in addition to LCA, the ileal proportion of CDCA was increased by both AMA interventions. CDCA and HCA are the most abundant BA produced by the porcine liver though a multistep reaction whose biosynthetic output is determined by the expression of genes encoding the enzymes CYP7A1 and CYP4A21^54^. Because the hepatic expression of these genes was not altered by ZAC and only HCA was decreased in colonic mucosa of AMA-treated pigs, it seems that the preferential use of this tri-hydroxylated BA (HCA) for the production of secondary BA by gut microbiota (rather than alterations in hepatic synthesis) dictated the observed changes in BA profiles. Noteworthy, this mechanism greatly differs from the previously described for antibiotics administered orally at therapeutic levels to rodents, which comprises suppression of BA transformations by intestinal microbes, in particular deconjugation and 7α-dehydroxylation, along with alterations in BA production by hepatocytes^22,45–47^.

Consistent with the notion that in rodents LCA and CDCA are the most potent endogenous agonists of the BA receptors TGR5 and FXR, respectively^20,34^, we next discovered that the AMA-induced alterations in BA profiles were associated with tissue-specific activation of pathways regulated by BA. In the intestines, FXR and TGR5 control signaling routes implicated in mucosal protection against inflammation, bacterial invasion, and dysregulated secretion of fluids and electrolytes^44,55,56^. It is important to note that early weaning, as performed in our experiments, has long-lived detrimental effects on such intestinal functions^41^. Concomitantly, both AMA combinations dampened the colonic expression of pro-inflammatory, NF-κB-dependent genes (*Ptgs2*, *Il8*) known the be controlled by FXR and TGR5^40,56^. Additionally, in colon ZAC induced genes regulated by FXR (*Ang1*, *Tgr5*) and TGR5 (*Gcg*, *Pcsk1)* that encode peptides responsible for preventing microbial infection (agiogenin)^28,42^ and maintaining the structural and functional integrity of the intestinal epithelium (GLP-2)^57,58^. Furthermore, ZCT attenuated permeability and secretory activity of ileal mucosa, pointing towards FXR-mediated inhibition of pathological paracellular transport^42^ and activity of Na^+^/K^+^ adenosine triphosphatase pumps as well as cystic fibrosis transmembrane conductance regulator channels^55^. Our findings not only agree with previously described enteroprotective effects of AMA^14–19^ and BA^59,60^ but also uncovered that they exert BA-mediated instructive functions in WAT and liver. The reciprocal changes in the expression of *Tgr5* and *Tnfα* in sWAT of ZCT-treated pigs along with the increased circulating levels of LCA and the immunosuppressive function of TGR5 in immune cells^44,56^, suggest that AMA triggered an anti-inflammatory process in WAT comprising down-regulation of the NF-κB-inflammatory pathway via LCA-mediated activation of TGR5 in WAT-resident macrophages. In addition to exert widespread immunomodulatory effects, we found that the alteration of BA signature by AMA also affected liver metabolism. In agreement with previous findings in mice^61^, the ability of ZAC to enhancing BA signaling through hepatic FXR resulted in counterregulatory effects on the expression of *Shp* and *Srebp1-c* and the consequential reduction in liver TAG. Certainly, this mechanism might have accounted for the reported repression of lipogenic genes in the liver of mice that were fed low-doses of antibiotics from weaning onwards^15^. Collectively, our results provide a mechanistic explanation for the increasingly recognized capacity of AMA of heightening the defense of intestinal mucosa and expand published data by showing that, irrespective of their bacterial spectrum, AMA target in a similar fashion the immune and metabolic homeostasis of tissues (WAT, liver) serving critical roles in the regulation of animal growth.

Further supporting the prediction that the non-therapeutic use of AMA entails alterations in BA signature and signaling, we demonstrated that both AMA interventions consistently increased the circulating concentration of FGF19. This emerging hormone is produced by the ileum in response to the BA-dependent activation of intestinal FXR^62^. Previous work showed that the enteral administration of CDCA to pigs enhances plasma FGF19^59^ and herein we proved that CDCA induces *Fgf19* expression in the porcine ileum more potently than HCA. Therefore, the opposing changes in the intestinal proportion of CDCA (increased) and HCA (decreased) caused by AMA likely colluded to promote the synthesis and release of FGF19 into circulation of medicated animals. Most biological functions of FGF19 are mediated by the interaction between FGFR4 and β-Klotho^62,63^. Expanding previous results from our group^64^, we showed that genes encoding these receptors are expressed at detectable levels in intestine, liver, WAT and muscle of pigs and that the abundance of their mRNA transcripts is not affected by AMA. Strikingly, our findings suggest that AMA intensify endocrine FGF19 signaling in tissues implicated in the regulation of growth. The main target of this enterokine is the liver, where FGF19 inhibits production of BA, glucose and fatty acids while stimulates synthesis of glycogen and protein^63,65^. Even though consistent alterations in the hepatic levels of BA and glycogen were not observed, the AMA-mediated changes in hepatic TAG (decreased) and protein (increased) suggest that amplified activity of the FGF19-FGFR4/β-Klotho pathway cooperated with the BA-FXR cascade to altering liver metabolism. In obese rodents, pharmacologic levels of FGF19 activate FGFR1/β-Klotho expressed in brain, liver, and WAT to cause weight loss and insulin sensitivity through increased energy expenditure^63,66^. We showed that the expression of genes involved in fatty acid oxidation and thermogenesis in sWAT were not affected by ZCT, which along the congruent gain in BW triggered by both medicinal treatments indicate that AMA does not promote FGF19 secretion to the extent required for activating such a mechanism. More important, recent evidence reveals that FGF19 promotes accretion of skeletal muscle mass through β-Klotho-dependent activation of the extracellular-signal-regulated protein kinase 1/2 and the ribosomal protein S6 kinase^37^. Because FGF19 acts upon the same signaling machinary to regulate protein synthesis in liver^63^, it seems reasonable to speculate that FGF19 is indeed a novel endocrine component of the growth-promoting action of AMA.

**Figure 7:**
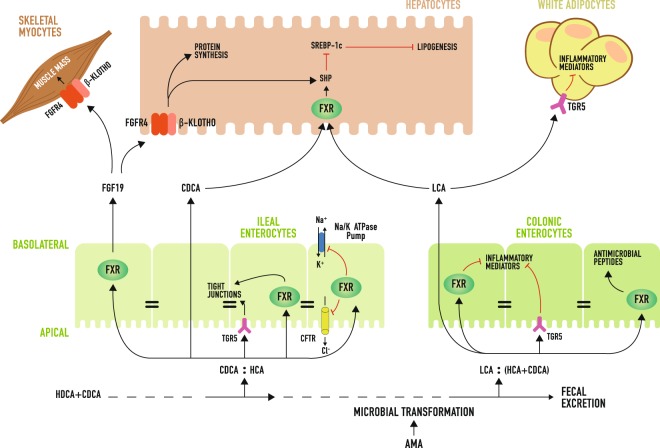
Model for bile acid (BA)-induced adaptations underlying growth promotion by antimicrobials (AMA) in pigs. The feeding of AMA to pigs promote the production of secondary BA by intestinal microbiota, resulting in tissue-specific increases in the proportion of BA that are most potent agonists of FXR and TGR5 (i.e., CDCA and LCA). As a result, BA signaling is amplified in ileum, colon, liver and WAT. Such activations lead to alterations in host intestinal physiology (i.e., reduced permeability and secretory activity), immunity (enhanced intestinal production of antimicrobial peptides, diminished inflammation in colon and WAT), and metabolism (decreased hepatic lipogenesis, heightened protein synthesis in liver and possibly in skeletal muscle). As a consequence, the immunometabolic demand for nutrients may be minimized thereby sparing nutrients for supporting animal growth. In addition, FGF19 may act as a hormone mediating the upregulation of protein synthesis in liver and skeletal muscle. Arrow head = induction, flat head = inhibition, CFTR = cystic fibrosis transmembrane receptor.

In summary, we demonstrate that combinations of AMA with different bacterial spectrum at doses commonly used for stimulating growth or preventing post-weaning enteritis in pigs promote the production of secondary BA by colonic microbiota (Fig. 7). In turn, this leads to tissue-specific increases in the proportion of BA that are most potent agonists of FXR and TGR5. As a consequence, BA signaling is amplified in intestine, liver, and WAT, which ultimately reinforces the protection of the intestinal mucosa against bacterial infection and pathological secretion of fluids and electrolytes, attenuates inflammation in colon and WAT, decreases hepatic lipogenesis, increases protein synthesis in liver, and stimulates the release of FGF19 into circulation. These alterations likely spare nutrients for growth by minimizing the nutritional cost associated with acute or chronic stimulation of the immune-inflammatory response resulting from challenging episodes during the lifetime of food-producing animals^67,68^. Additionally, we predict that FGF19 functions as an endocrine factor that improves the metabolic efficiency of AMA-treated animals by regulating energy and protein metabolism in liver and skeletal muscle. Certainly, more research is needed to confirm this hypothesis. This work identifies BA as integrators and modulators of the host physiological, metabolic, and immune-inflammatory responses to the AMA-mediated alterations in gut microbial metabolism, which makes them a promising target for the development of efficacious alternatives to AMA.

## Methods

Details are in Supplementary Methods

### Animal Experiments

All experimental procedures were approved by the Laboratory Animal Care Advisory Committee of the Faculty of Veterinary Sciences of the Universitat Autónoma de Barcelona, Spain and in accordance with the Guidelines for Animal Experimentation set by the same institution. A total of 120 Largewhite x Landrace x Pietrain, newly-weaned (22–23 days of age) pigs (60 of each sex) were obtained from the same commercial operation and used in two 35-day experiments conducted independently two years apart. In experiment 1, 72 piglets were distrusted into 12 pens (6 pigs/pen) and offered *ad libitum* access to water and feeds that were fed either untreated (CON; *n = *6) or medicated with zinc oxide, colistin, and amoxicillin (ZAC) at doses indicated in Supplementary Table S1. In experiment 2, 48 piglets were assigned to individual pens and offered *ad libitum* access to water and diets, which were fed either untreated (CON; *n = *24) or supplemented with zinc oxide, chlortetracycline, and tiamulin (ZCT) at doses shown in Supplementary Table S2. Starting at weaning, BW and feed intake were measured weekly in both experiments.

### Sample Collection

On day 34 and 35 of both experiments, 12 animals per group were selected and killed to collect samples of blood, intestinal contents, and tissues using procedures described in *Supplementary Methods*.

### Explant Assay

On day 34 of experiment 2, a portion of 10 cm from the mid-ileum of CON piglets was flushed with PBS 1 × , cut into 2–3 mm transversal slices and incubated in Dulbecco’s modified eagle medium containing the indicated concentrations of CDCA and/or HCA for 4 hours.

### Ussing Chamber Assay

On day 34 and 35 of experiment 2, ileal mucosa from the mid-ileum of CON and ZCT pigs were mounted in modified Ussing chambers to measure transepithelial electrical resistance (TER) and ion transport (*I*_sc_). Parameters were recorded every 10 seconds for a total of 2 h.

### Gut Microbiota Analysis

Samples of colonic content were processed to isolate bacterial DNA to assess the microbiome profile by massive sequencing of the hypervariable regions V1-V2 of the 16 S rRNA gene.

### Bile Acid Analysis

Bile acids were measured using UPLC-MS following procedures described by Li *et al*.^25^ with modifications detailed in *Supplementary Methods*.

### Bile Salt Hydrolase Assay

Enzyme activity was measured based on the generation of cholic acid-d5 after incubation of tauro-cholic acid-d5 (25 mM, Toronto Research Chemicals, Toronto, Canada) with proteins extracted from colonic contents (100 µg/mL) in 3 mM sodium acetate buffer (pH = 5.2). After 20 min incubation at 37 °C, the reaction was stopped with of IS solution (CDCA-d4) in acetonitrile and fast frozen in dry ice. Samples were analyzed using UPLC-MS as described for BA.

### Gene-Expression Analysis

Total RNA from homogenized tissues was isolated using a column affinity-based method (NucleoSpin RNA II; Macherey-Nagel, Düren, Germany). and transcribed into cDNA using High-Capacity cDNA Reverse Transcription Kit (Applied Biosystems/Life Technologies, Foster City, CA, USA). Quantitative real-time PCR was carried out using the specific porcine TaqMan Gene Expression Assays (Applied Biosystems, USA) or the primers pairs (Sigma-Aldrich, St. Louis, MO, USA) with SYBR Select Master Mix (Applied Biosystems, USA) specified in Supplementary Table S4.

### Hormone and Metabolite Quantification

FGF19 protein levels in plasma (100 µL) were assayed using a Pig Fibroblast Growth Factor 19 (FGF19) ELISA Kit (CSB-E17583p; Cusabio, China). FGF19 protein levels in liver and ileum were inmunodetected by western blot using primary 1/500 anti-FGF19 (ab85042, Abcam Plc, Cambridge, UK) antibody and 1/5000 anti-β-actin (A5441, Sigma-Aldrich) antibody for loading normalization. Commercially available kits and reagents were used to assess plasma insulin (AKRIN-013T; Shibayagi Co., Ltd., Shibukawa, Japan), adiponectin (RD591023200R; BioVendor R&D, Brno, Czech Republic), leptin (026475; US Biological, Swampscott, MA, USA), glucose (G3293; Sigma-Aldrich), non-esterified fatty acids (NEFA) (434–91795, 436–91995; Wako Chemicals GmbH, Neuss, Germany), and triglycerides (TR0100; Sigma-Aldrich). Glycogen, triglycerides and protein content in liver, were measured in homogenized tissue using the Glycogen Assay Kit (MAK016; Sigma-Aldrich), the Serum Triglyceride Determination Kit (TR0100; Sigma-Aldrich) or the BCA assay, respectively.

### Statistical Analysis

Animal performance parameters including feed consumption and body weight gain were analyzed using a mixed-effect model with repeated measures in time (week). In the model, pen (experiment 1) or pig (experiment 2) nested within treatment were entered as random variables and treatment, time and their two-way interaction were considered as fixed effects. The same mixed-model with pig as the experimental unit but without repeated measures was used to analyze concentration of bile acids, hormones, and metabolites as well as bile salt hydrolase activity, transepithelial electrical resistance, and short circuit current. Gene expression data were analyzed using Student t test. Statistical analyses were performed with SAS (release 9.2, SAS Institute). Microbial raw sequencing reads were demultiplexed, quality-filtered and analyzed using QIIME 1.9.1^69^.

## Electronic supplementary material

Supplementary Information

## Data Availability

The datasets generated during and/or analyzed during the current study are available from the corresponding author on reasonable request.
