# Supplementary material for: Antimicrobial promotion of pig growth is associated with tissue-specific remodeling of bile acid signature and signaling
Source: Sci Rep. 2018 Sep 12;8:13671. doi: 10.1038/s41598-018-32107-9 (PMC6135865; doi:10.1038/s41598-018-32107-9)

**Supplementary Information**

**Antimicrobial promotion of pig growth is associated with tissue-specific remodeling of bile acid signature and signaling**

Ignacio R. Ipharraguerre^a,b*^, Jose J. Pastor^b^, Aleix Gavaldà-Navarro^c^, Francesc Villarroya^c^, and Alessandro Mereu^b,1^

^a^Institute of Human Nutrition and Food Science, University of Kiel, Hermann-Rodewald-Strasse 6-8, D-24128 Kiel, Germany

^b^Innovation Division, Lucta S.A., Parc de Recerca UAB, Edifici Eureka, 08193 Bellaterra, Catalonia, Spain

^c^Departament de Bioquímica i Biomedicina Molecular, Institut de Biomedicina (IBUB), Universitat de Barcelona, and CIBER Fisiopatología de la Obesidad y Nutrición, Avinguda Diagonal 645, Edifici nou Pl.-1 , 08028 Barcelona, Catalonia, Spain

***Corresponding author**: Ignacio R. Ipharraguerre; Institute of Human Nutrition and Food Science, University of Kiel, Hermann-Rodewald-Strasse 6-8, D-24128 Kiel, Germany; +34 625 95 14 21; [ipharraguerre@foodsci.uni-kiel.de](mailto:ipharraguerre@foodsci.uni-kiel.de)

^1^**Current address**: Yara Iberian, C/ Infanta Mercedes 31 – 2^nd^ floor, 28020 Madrid, Spain

**Supplementary Methods**

**Animals and Housing.** All experimental procedures were approved by the Laboratory Animal Care Advisory Committee of the Faculty of Veterinary Sciences of the Universitat Autónoma de Barcelona, Spain. A total of 120 domestic pigs (60 of each sex) from the commercial operation and breed (Largewhite x Landrace x Pietrain) were used in two 35-day experiments. conducted at the Swine Experimental Unit of Lucta S.A. (Girona, Spain). In experiment 1 (Exp.1), 72 piglets (36 of each sex) were weaned at 22 ± 2 days of age weighing 6.4 ± 0.7 kg and distributed into 12 pens (6 pigs/pen) balanced by body weight (BW) and sex (50:50 male to female ratio). Piglets were offered *ad libitum* access to water, pre-starter (day 1 to 14 of study) and starter (day 15 to 35 of study) feeds that were fed either untreated (CON; *n* = 6) or medicated with zinc oxide, colistin, and amoxicillin (ZAC) at doses indicated in Table S1. In experiment 2 (Exp.2), 48 piglets (24 of each sex) were weaned at 23 ± 2 days of age weighing 5.8 ± 0.8 kg, assigned to 48 individual pens and offered *ad libitum* access to water, pre-starter (day 1 to 14 of study) and starter (day 15 to 35 of study) diets, which were fed either untreated (CON; *n* = 24) or supplemented with zinc oxide, chlortetracycline, and tiamulin (ZCT) at doses shown in Table S2. Starting at weaning, BW and feed intake were measured weekly in both experiments.

**Sample Collection.** In Exp.1 and Exp.2, 12 animals were selected based on BW (closest to the corresponding group median) to collect samples of blood, intestinal contents, and tissues using the same procedures on day 34 and 35 of each study. After 2-3 h of feed deprivation, blood samples were first obtained via jugular venipuncture. For plasma collection blood samples were placed into tubes containing EDTA and aprotinin (BD Vacutainer®) and held in ice-cold water for 30 min, whereas for serum collection samples were placed into tubes containing spray-coated silica (BD Vacutainer®) and held at 4 °C for 15 min. Samples were centrifuged at 4,000×g for 10 min and stored at –80ºC until later analysis. Following blood sampling, animals were killed by captive bolt and exsanguinated. The abdomen was opened, and the liver, kidneys, and intestines were removed. The intestines were dissected into sections designated as ileum (from the first Peyer’s patch to the ileocecal valve) and colon (30 cm caudally from the ileocecal valve to the rectum). Colonic contents were immediately collected into sterile vials, snap-frozen in liquid N, and stored at -80ºC until microbiota analysis. A 10-cm segment was removed from the mid-section of the ileum and colon, opened longitudinally, and flushed with saline solution. Mucosal scrapings taken from half of these samples along with samples of liver (right lobe), skeletal muscle (biceps femoris), subcutaneous and visceral (kidneys) fat were placed in RNA*later*^®^ (Ambion, USA) and stored at -80ºC until gene expression analysis. Two other sets of samples from the same tissues and locations were span-frozen in liquid N and stored at -80ºC for later analysis.

**Explant Assay.** On day 34 of Exp.2, ileal sections from 4 pigs of the control (CON) group were dissected (from the first Peyer’s patch to the ileocecal valve) and a portion of 10 cm from the mid-section was selected and flushed with PBS 1x using a syringe to remove the luminal contents. Transversal slices of the ileum (2-3 mm) were obtained and placed in 35 mm cell culture dishes, containing 3 ml of Dulbecco’s Modified Eagle Medium, 20 mmol/l glucose, 10% FBS, 100 U/ml penicillin, and 100 μg/ml streptomycin (Gibco Life Technologies, Foster City, CA, USA). In addition, slices were treated with 300 µM of chenodeoxycholic acid or hyocholic acid, or with 3000 µM of chenodeoxycholic acid and increasing concentrations of hycholic (0, 30, 300 and 3000 µM) (Steraloids Inc., Newport, USA). Ileal explants were maintained in an incubator with a humidified atmosphere of 5% CO2 at 37°C for 4 hours. After incubation, ileal explants were removed from media and immediately frozen in liquid nitrogen and stored at -80ºC until processing for RNA isolation and gene expression analysis.

**Ussing Chamber Assay.** On day 34 and 35 of Exp.2, 10-cm long ileal samples were washed with cold PBS (pH = 7.4), opened longitudinally through its mesenteric side and placed in ice-cold ringer buffer (Na+ = 154 mmol/L, K+ = 6.3 mmol/L, Cl- = 137 mmol/L, H2PO4- = 0.3 mmol/L, Ca2+ = 1.2 mmol/L, Mg2+ = 0.7 mmol/L, HCO3- = 24 mmol/L, pH = 7.4) gassed with carbogen (95% O_2_, 5% CO_2_). The muscle layer was carefully removed, the mucosa fixed in 1 cm^2^ inserts and mounted in modified Ussing chambers (Physiologic Instruments, San Diego, CA, USA). Intestinal mucosa was bathed with 5 mL of ringer buffer plus 10 mmol/L mannitol at the luminal side and 5 mL of ringer buffer with 10 mmol/L glucose at the basolateral side, with constant oxygenation with carbogen mixture at both sides. Transepithelial electrical resistance (TER) and short-circuit current (*I*_sc_) were recorded every 10 seconds for 2 h, after a 30 min period of equilibration. A total of 16 pigs (8 from each group) were used in 8 independent assays (*n* = 8). In each assay, 4 chambers were randomly assigned to one animal of each group (CON vs ZCT).

**Gut Microbiota Analysis.** Immediately after thawing, samples of colonic content were processed to isolate bacterial DNA to assess the microbiome profile by massive sequencing of the hypervariable regions of the 16S rRNA gene. Amplicons of the V1-V2 16S rRNA region were amplified with barcoded forward primer F27 and reverse primer R338, with sequencing adaptors at the 5′ end. Concentration and quality were determined using Agilent Bioanalyzer 2100 (Agilent Technologies, Waldbronn, Germany) for each amplicon. Samples were sequenced on an Ion Torrent Personal Genome Machine (PGM) with the Ion 318 Chip Kit v2 (Life Technologies) under manufacturer’s conditions.

**Bile Acid Analysis.** Determination of bile acids was performed as described by Li et al.^1^ with modifications. Analyses were performed in an Acquity UPLC (Waters Corp. Mildford, MA) connected to a Xevo G2 Qtof mass spectrometer (Waters Corp.) operating in full scan negative mode. Chromatographic separation was achieved using a reverse-phase column (Acquity BEH C18 2.1 x 100 mm, 1.7 µm) and mobile phases comprising water + 0.1% formic acid (A) and acetonitrile + 0.1% formic acid (B). Flow rate was set to 0.5 mL/min and oven temperature to 40°C. Stepwise gradient was programmed as follows: *0 min*, 15% A; *12 min*, 40% B; *16 min*, 60% B; *19 min*, 85% B; *19.5 min*, 100% B. MS conditions were: *Ionization mode* = ES(-); *capillary* = 0.5 kV; *cone* = 30 V; *extraction cone* = 4 V, *source temperature* = 120°C; *desolvation temperature* = 350°C; *gas flow* = 900 L/h; *mass range* = 100 – 1200 m/z. Leucine-enkephalin (2 ng/mL) was used as lock mass. Data processing was performed with QuanLynx software (Waters Corp.) and quantification performed by ion areas based on standard curves using authentic standards and chenodeoxycholic acid-d4 as internal standard (IS). Sample preparation was adapted to the bile acid concentration in tissues/fluids. Protein in plasma samples (100 µL) was precipitated with 300 µL of IS solution in acetonitrile. After mixing and centrifugation (15.000 g x 10 min, 4°C) supernatant was collected and completely evaporated in a MiVac DNA centrifugal vacuum concentrator (Genevac, Ipswich, UK). Solid was reconstituted with 100 µL of H_2_O/MeOH 1:1 and directly injected in the UPLC. Intestinal samples (100 mg) were extracted with either 1 mL (ileum) or 750 µL (colon) of IS solution in H_2_O/ACN 1:1, centrifuged (15.000 x 15 min, 4°C) and supernatant properly diluted in H_2_O/ACN 2:3 for UPLC analysis. Liver samples (50 mg) were also extracted with 800 µL of IS solution in H_2_O/ACN 1:1 and diluted in H_2_O/ACN 2:3 for chromatographic analysis. In all cases, 4 independent replicates per sample were prepared and analyzed.

**Bile Salt Hydrolase Assay.** Proteins were extracted from 100 mg of colonic samples using 1 mL of PBS (pH = 7.4) and freeze – thaw cycles. Enzyme activity was measured based on the generation of cholic acid-d5 after incubation of tauro-cholic acid-d5 (Toronto Research Chemicals, Toronto, Canada) with proteins extracted from colonic contents in proper conditions. Incubation was carried out in 3 mM sodium acetate buffer (pH = 5.2) at fixed protein concentration of 100 µg/mL, tauro-cholic acid-d5 concentration of 25 mM and final volume of 200 µL. After 20 min incubation at 37°C, a 25 µL sample was mixed with 25 µL of IS solution (chenodeoxycholic acid-d4) in acetonitrile and frozen in dry ice. Cholic acid-d5 was measured using UPLC-MS as described for other bile acids. The concentration of cholic acid-d5 was calculated based on standard curve using authentic standard. Four independent replicates per sample were prepared and analyzed.

**Gene-Expression Analysis.** Dissected tissues (ileal explants, ileal mucosa, colonic mucosa, liver, white adipose tissue, and skeletal muscle) were homogenized using a TissueLyser LT (Qiagen, Germany). Total RNA from homogenized tissues was isolated using a column affinity-based method (NucleoSpin RNA II; Macherey-Nagel, Düren, Germany). Total RNA (500 ng) was transcribed into cDNA using High-Capacity cDNA Reverse Transcription Kit (Applied Biosystems/Life Technologies, Foster City, CA, USA). For quantitative analysis of mRNA expression, quantitative real-time polymerase chain reaction (qPCR) was performed on a 7500 Real-Time PCR System (Applied Biosystems, USA) using the specific porcine TaqMan Gene Expression Assays (Applied Biosystems, USA) or the primers pairs (Sigma-Aldrich, St. Louis, MO, USA) with SYBR Select Master Mix (Applied Biosystems, USA) specified in Table S4. Relative mRNA levels of target genes were normalized with respect to that of 18S rRNA (Hs99999901_s1), using the comparative (2-ΔCT) method. Transcript levels were considered undetectable in cases where the CT value was >40 under our experimental conditions.

**FGF19 Protein Quantification.** For the quantification of the FGF19 protein levels in plasma, 100 µL of plasma were assayed using a Pig Fibroblast Growth Factor 19 (FGF19) ELISA Kit (CSB-E17583p; Cusabio, China), following the recommendations of the manufacturer. FGF19 protein levels in liver and ileum were inmunodetected by western blot. Briefly, dissected tissues were homogenized using an TissueLyser LT (Qiagen, Germany) in lysis buffer (50 mmols/l Tris-HCl pH 7.4, 150 mmols/l NaCl, 1.5 mmols/l MgCl2, 1 mmols/l EDTA, 1 mmols/l EGTA, 40 mmols/l β-glycerophosphate, 2 mmols/l Na3VO4, 1 mmols/l PMSF, 1 mmols/l DTT, 1% Igepal CA-630) containing complete protease inhibitor cocktail (Roche Applied Science). Lysates were centrifuged at 1,500 x g for 5 minutes at 4°C to remove intact cells, and protein content was quantified using the BCA assay. For Western blotting, proteins (40 µg) were resolved by SDS-PAGE and transferred to a PVDF membrane (Immobilon; Millipore). Membranes were exposed to primary 1/500 anti-FGF19 (ab85042, Abcam Plc, Cambridge, UK) and 1/5000 anti-β-actin (A5441, Sigma-Aldrich) antibodies, diluted in 1x PBS containing 0.1% Tween-20, following the recommendations of the manufacturer. Signals were detected using enhanced chemiluminescence HRP substrate (Millipore) and analyzed with a Luminescent Image Analyzer LAS-3000 (Fujifilm Life Science, Tokyo, Japan). Signal intensities were quantified using Multi Gauge software (Fujifilm).

**Hormone and Metabolite Quantification.** Commercially available kits and reagents were used to assess plasma insulin (AKRIN-013T; Shibayagi Co., Ltd., Shibukawa, Japan), adiponectin (RD591023200R; BioVendor R&D, Brno, Czech Republic), leptin (026475; US Biological, Swampscott, MA, USA, glucose (G3293; Sigma-Aldrich), non-esterified fatty acids (NEFA) (434-91795, 436-91995; Wako Chemicals GmbH, Neuss, Germany), and triglycerides (TR0100; Sigma-Aldrich). For quantification of glycogen in liver, 10-15 mg of liver were homogenized using a TissueLyser LT in 300 µl of distilled water, boiled for 10 minutes and centrifuged at 13.000 xg for 5 minutes. Glycogen was measured in the supernatant using the Glycogen Assay Kit (MAK016; Sigma-Aldrich). For quantification of triglycerides in liver, 20-30 mg of liver were homogenized using a TissueLyser LT in 400 µl of distilled water containing 5% Igepal CA-630, boiled during 5 minutes twice, centrifuged at 13.000 xg for 5 minutes. Triglycerides were measured in the supernatant using the Serum Triglyceride Determination Kit (TR0100; Sigma-Aldrich). For measuring protein content in liver, 20-25 mg of liver were homogenized using an TissueLyser LT (Qiagen, Germany) in 500 µl of lysis buffer (described above), centrifuged at 13.000 xg for 5 minutes and quantified using the BCA assay. In all quantifications, values were normalized by tissue weights used for the homogenizations.

**Statistical Analysis.** Animal performance parameters, including average daily feed intake (FI) and body weight (BW) gain, were analyzed using a mixed-effect model with repeated measures in time (week). In the model, pen (FI in Exp.1) or pig (BW in Exp.1, FI and BW in Exp.2) nested within treatment were entered as random variables and treatment, time and their two-way interaction were considered as fixed effects. The smallest value for the Akaike’s information criterion was used to identify the most appropriate covariance structure. The same mixed-model with pig as the experimental unit but without repeated measures was used to analyze concentration of bile acids, hormones, and metabolites as well as bile salt hydrolase activity, transepithelial electrical resistance, and short circuit current. Model diagnostics included testing for a normal distribution of the error residuals and homogeneity of variance. Least squares means were separated into significant effects using Fisher’s LSD. Gene expression data were analyzed using Student t test.  Statistical analyses were performed with SAS (release 9.2, SAS Institute). Microbial raw sequencing reads were demultiplexed, quality-filtered and analyzed using QIIME 1.9.1^2^. Quality-filtered reads were clustered into operational taxonomic units (OTUs) for taxonomy analyses. Taxonomic assignment of representative OTUs was performed using the RDP Classifier^3^. Alignment of sequences was performed using PyNast^4^ as default in QIIME pipeline, with an extra filtering step in aligned and taxonomy-assigned OTU table to filter-out sequences that represent less than 0.005% of total OTUs. Downstream analyses were performed at the same depth per sample to standardize for unequal sequencing depth of the samples. Alpha diversity (within group) was assessed using the Shannon index, whose statistical significance was determined with 999 permutations using the non-parametric Monte Carlo permutation test. Beta diversity (between groups) were analyzed using Weighted and Unweighted UniFrac (ANOSIM).

**Supplementary References**

1. Li, F. et al. Microbiome remodelling leads to inhibition of intestinal farnesoid X receptor signalling and decreased obesity. *Nat. Commun.* **4**, 2384 (2013).

2. Caporaso, J. G. et al. QIIME allows analysis of high-throughput community sequencing data. *Nat. Methods* **7**, 335–336 (2010).

3. Wang, Q., Garrity, G. M., Tiedje, J. M. & Cole, J. R. Naïve Bayesian classifier for rapid assignment of rRNA sequences into the new bacterial taxonomy. *Appl Environ Microbiol* **73**, 5261–5267 (2007).

4. Caporaso, J. G. et al. PyNAST: A flexible tool for aligning sequences to a template alignment. *Bioinformatics* **26**, 266–267 (2010).

**Supplementary Table S1. Composition of diets fed to pigs in experiment 1^1^.**

|  | | Feeding period | | |
| --- | --- | --- | --- | --- |
|  | | 0-14 days post-weaning |  | 15-35 days post-weaning |
| *Ingredient (g/kg as fed basis)* | |  |  |  |
| Corn | | 319 |  | 205 |
| Wheat | | 135 |  | 339 |
| Barley | | 64 |  | 100 |
| Lactose | | 86 |  | 36 |
| Heated full-fat soybeans | | 200 |  | 90 |
| Processed soybean meal^2^ | | 120 |  | 25 |
| Soybean meal (44% CP) | | - |  | 148 |
| Soybean oil | | 34 |  | 17 |
| Calcium carbonate | | 10.8 |  | 8.3 |
| Monocalcium phosphate | | 12.8 |  | 13.0 |
| Trace elements and vitamin premix^3^ | | 4.0 |  | 4.0 |
| Salt | | 2.0 |  | 2.0 |
| L-Lysine-HCl | | 5.0 |  | 5.65 |
| DL-Methionine | | 2.2 |  | 1.9 |
| L-Threonine | | 2.0 |  | 2.1 |
| L-Tryptophan | | 0.30 |  | 0.15 |
| *Antimicrobials (g/kg as fed basis)* | |  |  |  |
| Zinc oxide | | 2.5 |  | 2.5 |
| Amoxicillin thrihydrate | | 0.3 |  | 0.3 |
| Colistin sulfate | | 0.12 |  | 0.12 |
| *Calculated nutrient composition* *(% as fed basis)* | | |  |  |
| Crude protein | 19.4 | |  | 18.5 |
| Digestible amino acids^4^ |  | |  |  |
| Lysine | 1.39 | |  | 1.32 |
| Methionine | 0.50 | |  | 0.46 |
| Methionine + cysteine | 0.83 | |  | 0.77 |
| Threonine | 0.90 | |  | 0.85 |
| Tryptophan | 0.26 | |  | 0.23 |
| Metabolizable energy (MJ/kg) | 14.8 | |  | 13.8 |

^1^Diets were fed either without antimicrobials to control pigs (CON) or with antimicrobials to treated pigs (designated as ZAC).

^2^HP300 (Hamlet Protein A/S, Horsens, Denmark)

^3^Containing the following: vitamin A, 10000 UI; vitamin D_3_, 2000 UI; vitamin E (alfa‑tocopherol), 25 mg; vitamin B_1_, 1.5 mg; vitamin B_2_, 3.5 mg; vitamin B_6_, 2.4 mg; vitamin B_12_, 20 µg; vitamin K_3_, 1.5 mg; calcium panthotenate, 14 mg; nicotinic acid, 20 mg; folic acid, 0.5 mg; biotin, 50 µg; iron sulfate, 120 mg; calcium iodate, 0.75 mg; cobalt carbonate, 0.6 mg; copper sulfate, 150 mg; magnesium oxide, 60 mg; zinc oxide, 110 mg; sodium selenite, 0.37 mg.

^4^Ileal standardized digestibility.

**Supplementary Table S2. Composition of diets fed to pigs in experiment 2^1^.**

|  | | Feeding period | | |
| --- | --- | --- | --- | --- |
|  | | 0-14 days post-weaning |  | 15-35 days post-weaning |
| *Ingredient (g/kg as fed basis)* | |  |  |  |
| Corn | | 348 |  | 200 |
| Wheat | | 112 |  | 300 |
| Barley | | 80 |  | 146 |
| Heated full-fat soybeans | | 148 |  | 80 |
| Processed soybean meal^2^ | | 42 |  | - |
| Soybean meal (47% CP) | | - |  | 122 |
| Soybean oil | | 40 |  | 24 |
| Sweet whey milk powder | | 126 |  | 50 |
| Fish meal | | 70 |  | 40 |
| Calcium carbonate | | 8.5 |  | 9.2 |
| Monocalcium phosphate | | 6.7 |  | 8.4 |
| Trace elements and vitamin premix^3^ | | 4.0 |  | 4.0 |
| Salt | | - |  | 2.0 |
| L-Lysine-HCl | | 3.6 |  | 4.0 |
| DL-Methionine | | 1.6 |  | 1.3 |
| L-Threonine | | 1.3 |  | 1.4 |
| L-Tryptophan | | 0.4 |  | 0.1 |
| Preservatives and flavors | | 5.0 |  | 5.0 |
| *Antimicrobials (g/kg as fed basis)* | |  |  |  |
| Zinc oxide | | 2.5 |  | 2.5 |
| Tiamulin | | 0.04 |  | 0.04 |
| Chlortetracycline | | 0.4 |  | 0.11 |
| *Calculated nutrient composition* *(% as fed basis)* | | |  |  |
| Crude protein | 19.5 | |  | 19.1 |
| Digestible amino acids^4^ |  | |  |  |
| Lysine | 1.39 | |  | 1.30 |
| Methionine | 0.54 | |  | 0.45 |
| Methionine + cysteine | 0.84 | |  | 0.77 |
| Threonine | 0.90 | |  | 0.84 |
| Tryptophan | 0.26 | |  | 0.23 |
| Digestible energy (MJ/kg) | 14.9 | |  | 14.0 |

^1^Diets were fed either without antimicrobials to control pigs (CON) or with antimicrobials to treated pigs (designated as ZAC).

^2^HP300 (Hamlet Protein A/S, Horsens, Denmark)

^3^Containing the following: vitamin A, 10000 UI; vitamin D_3_, 2000 UI; vitamin E (alfa‑tocopherol), 25 mg; vitamin B_1_, 1.5 mg; vitamin B_2_, 3.5 mg; vitamin B_6_, 2.4 mg; vitamin B_12_, 20 µg; vitamin K_3_, 1.5 mg; calcium panthotenate, 14 mg; nicotinic acid, 20 mg; folic acid, 0.5 mg; biotin, 50 µg; iron sulfate, 120 mg; calcium iodate, 0.75 mg; cobalt carbonate, 0.6 mg; copper sulfate, 150 mg; magnesium oxide, 60 mg; zinc oxide, 110 mg; sodium selenite, 0.37 mg.

^4^Ileal standardized digestibility.

**Supplementary Table S3. Gut microbiota beta diversity (between individuals) of piglets fed antimicrobials for 35 days following weaning.**

| **Analysis of similarities (ANOSIM)^1^** | | |
| --- | --- | --- |
|  | Unweighted UniFrac | Weighted UniFrac |
| **ZAC vs. CON** |  |  |
| Sample size | 20 | 20 |
| *R* | 0.555 | 0.391 |
| *P-value* | 0.001 | 0.001 |
|  |  |  |
| **ZCT vs. CON** |  |  |
| Sample size | 24 | 24 |
| *R* | 0.081 | 0.066 |
| *P-value* | 0.101 | 0.106 |
| ^1^Analyses were conducted with QUIIME (2); CON = control group of pigs fed cereal-based diets; ZAC = treated group of pigs fed same diet as CON but medicated with zinc oxide, amoxicillin, and colistin sulfate; ZCT = treated group of pigs fed same diet as CON but medicated with zinc oxide, chlortetracycline, and tiamulin. | | |

**Supplementary Table S4. Primers used for quantitative reverse transcription PCR**

| **SYBR Green Technology** | |  |
| --- | --- | --- |
| **Gene** | **Accession No.** | **Primers (5’ – 3’)** |
| *FGF19* | NC_010444.4 | F: AAGATGCAAGGGCAGACTCA |
|  |  | R: AGATGGTGTTTCTTGGACCAGT |
| *NR1H4 (FXR)* | NC_010447.5 | F: TTTGTGTCGTTTGCGGAGAG |
|  |  | R: GTTGCCCCCATTTTTACACTTG |
| *GPBAR1 (TGR5)* | NC_010457.5 | F: CACCCATCAGGACACCAGAC |
|  |  | R: AGGGGAAGGGCTACTCACAT |
| *NR0B2 (SHP)* | NC_010448.4 | F: GCCTACCTGAAAGGGACCAT |
|  |  | R: CAACGGGTGTCAAGCCTTTA |
| *KLB* | NC_010450.4 | F: ATCGACGACCAGTCTCTGGA |
|  |  | R: AGGGAAGCCATTGTTGCTGA |
|  |  |  |
| **TaqMan Technology** | |  |
| **Gene** | **Accession No.** | **Assay ID** |
| *ANG1* | NM_001044573.2 | Ss03380513_u1 |
| *CCL2 (MCP-1)* | NM_214214.1 | Ss03394377_m1 |
| *CYP27A1* | NM_001243304.1 | Ss03377319_u1 |
| *CYP4A21* | NM_214425.1 | Ss03384826_u1 |
| *CYP7A1* | NM_001005352.2 | Ss03378689_u1 |
| *DIO2* | NM_001001626.1 | Ss03391525_m1 |
| *FGFR1IIIc* | AJ577088.1 | Ss03373552_s1 |
| *FGFR4* | AM778831.1 | Ss03376194_u1 |
| *IL-8* | NM_213867.1 | Ss03392435_m1 |
| *IL-18* | NM_213997.1 | Ss03391203_m1 |
| *LPL* | NM_214286.1 | Ss03394605_m1 |
| *PCSK1* | NM_214038.1 | Ss03393661_s1 |
| *PPARg* | NM_214379.1 | Ss03394829_m1 |
| *PPARGC1* | NM_213963.1 | Ss03393114_u1 |
| *PTGS2 (COX2)* | NM_214321.1 | Ss03394692_m1 |
| *SLC2A4 (GLUT4)* | AB005285.1 | Ss03372899_u1 |
| *SREBF1 (SREBP1-c)* | NM_214157.1 | Ss03382914_u1 |
| *TNFa* | NM_214022.1 | Ss03391318_g1 |
| *UCP3* | NM_214049.1 | Ss03391403_m1 |

F = forward; R = reverse

**Supplementary Figure S1. Balance between total bile salts and acids in ileal mucosa of piglets fed antimicrobials.** Two independent cohorts of piglets from the same commercial operation were weaned averaging 23 ± 2 days of age and fed for 35 days cereal-based diets medicated with combinations of antimicrobials, which were either zinc oxide, amoxicillin, and colistin (ZAC) or zinc oxide, chlortetracycline, and tiamulin (ZCT) at dosing levels routinely used for preventing bacterial enteritis and stimulating pig growth. Control pigs (CON) received no antimicrobials. The concentration of bile salts and acids was measured via UPLC-MS in samples taken on days 34 and 35. Data from the two experiments were combined in the figure. (*A*) Proportion (%) of bile salts and acids in the ileal mucosa of pigs fed CON or ZAC. (*B*) Proportion of bile salts and acids in the ileal mucosa of pigs fed CON or ZCT. Data were analyzed with ANOVA. Least squares means ± SEM are plotted, *n* = 12.

**Supplementary Figure S2. Concentration of total bile acids in the enterohepatic system and systemic circulation of piglets fed antimicrobials.** Two independent cohorts of piglets from the same commercial operation were weaned averaging 23 ± 2 days of age and fed for 35 days cereal-based diets medicated with combinations of antimicrobials, which were either zinc oxide, amoxicillin, and colistin (ZAC) or zinc oxide, chlortetracycline, and tiamulin (ZCT) at dosing levels routinely used for preventing bacterial enteritis and stimulating pig growth. Control pigs (CON) received no antimicrobials. The concentration of bile acids and salts was measured via UPLC-MS in samples taken on days 34 and 35. Data from the two experiments were combined in the figure. (*A*) Total bile acids in liver, plasma, and intestine of pigs fed CON or ZAC. (*B*) Total bile acids in liver, plasma, and intestine of pigs fed CON or ZCT. Data were analyzed with ANOVA. Least squares means ± SEM are plotted, ^*^*P* < 0.05, ^**^*P* < 0.01, *n* = 12.

**Supplementary Figure S3. Expression of FGF19 receptor genes in liver and adipose tissue of piglets fed antimicrobials.** Two independent cohorts of piglets from the same commercial operation were weaned averaging 23 ± 2 days of age and fed for 35 days cereal-based diets medicated with combinations of antimicrobials, which were either zinc oxide, amoxicillin, and colistin sulfate (ZAC) or zinc oxide, chlortetracycline, and tiamulin (ZCT) at dosing levels routinely used for preventing bacterial enteritis and stimulating pig growth. Control pigs (CON) received no antimicrobials. Gene expression was measured by qRT-PCR in samples taken on days 34 and 35. Data from the two experiments were combined in the figure. (*A*) mRNA abundance of *Fgfr4* and *Klb* (β-Klotho) in the liver of pigs fed CON or ZAC. (*B*) mRNA abundance of *Fgfr4*, *Fgfr1IIIc*, and *Klb* in visceral (kidney) adipose tissue of pigs fed CON or ZCT. Data were analyzed with Student t test. Means ± SEM are plotted, *n* = 9-12.

**Supplementary Figure S4. Expression of genes responsive to bile acids in colonic mucosa of piglets fed antimicrobials.** Two independent cohorts of piglets from the same commercial operation were weaned averaging 23 ± 2 days of age and fed for 35 days cereal-based diets medicated with combinations of antimicrobials, which were either zinc oxide, amoxicillin, and colistin (ZAC) or zinc oxide, chlortetracycline, and tiamulin (ZCT) at dosing levels routinely used for preventing bacterial enteritis and stimulating pig growth. Control pigs (CON) received no antimicrobials. Gene expression was measured by qRT-PCR in intestinal mucosa samples taken on days 34 and 35. Data from the two experiments were combined in the figure. mRNA abundance of *Ang1* (*A* and *E*), *Ptgs1* (*B* and *F*), *Gcg* (*C* and *G*), and *Pcsk1* (*D* and *H*) in colonic mucosa of pigs fed ZAC (*A-D*) or ZCT (*E*-*H*). Data were analyzed with Student t test. Means ± SE are plotted, ^*^*P* < 0.05, ^**^*P* < 0.01, ^***^*P* < 0.001, *n* = 8-12.

**Supplementary Figure S5. Expression of genes involved in thermogenesis and fatty acid oxidation in subcutaneous white adipose (sWAT) tissue of piglets fed antimicrobials.** Piglets were weaned averaging 23 ± 2 days of age and fed for 35 days cereal-based diets medicated with a combination of zinc oxide, chlortetracycline, and tiamulin (ZCT) at dosing levels routinely used for stimulating pig growth. Control pigs (CON) received no antimicrobials. Gene expression was measured by qRT-PCR in sWAT samples taken on days 34 and 35. mRNA abundance of *Dio2* (*A*), *Ucp3* (*B*), *Pgc1α* (*C*), *Pparγ* (*D*), *Lpl* (*E*), and *Glut4* (*F*). Data were analyzed with Student t test. Means ± SEM are plotted, *n* = 11-12.

**Supplementary Figure S6. Expression of genes involved in thermogenesis and fatty acid oxidation in skeletal muscle of piglets fed antimicrobials.** Piglets were weaned averaging 23 ± 2 days of age and fed for 35 days cereal-based diets medicated with a combination of zinc oxide, chlortetracycline, and tiamulin (ZCT) at dosing levels routinely used for stimulating pig growth. Control pigs (CON) received no antimicrobials. Gene expression was measured by qRT-PCR in skeletal muscle samples taken on days 34 and 35. Transcript level of *Tgr5* (*A*), *Dio2* (*B*), *Ucp3* (*C*), *Pgc1α* (*D*), *Pparα* (*E*), *Lpl* (*F*), and *Glut4* (*G*). Data were analyzed with Student t test. Means ± SE are plotted, *n* = 10-12.

**Supplementary Figure S7. Full-length blots for FGF19 and β-actin immunodetection in ileum of pigs fed CON or ZAC.**


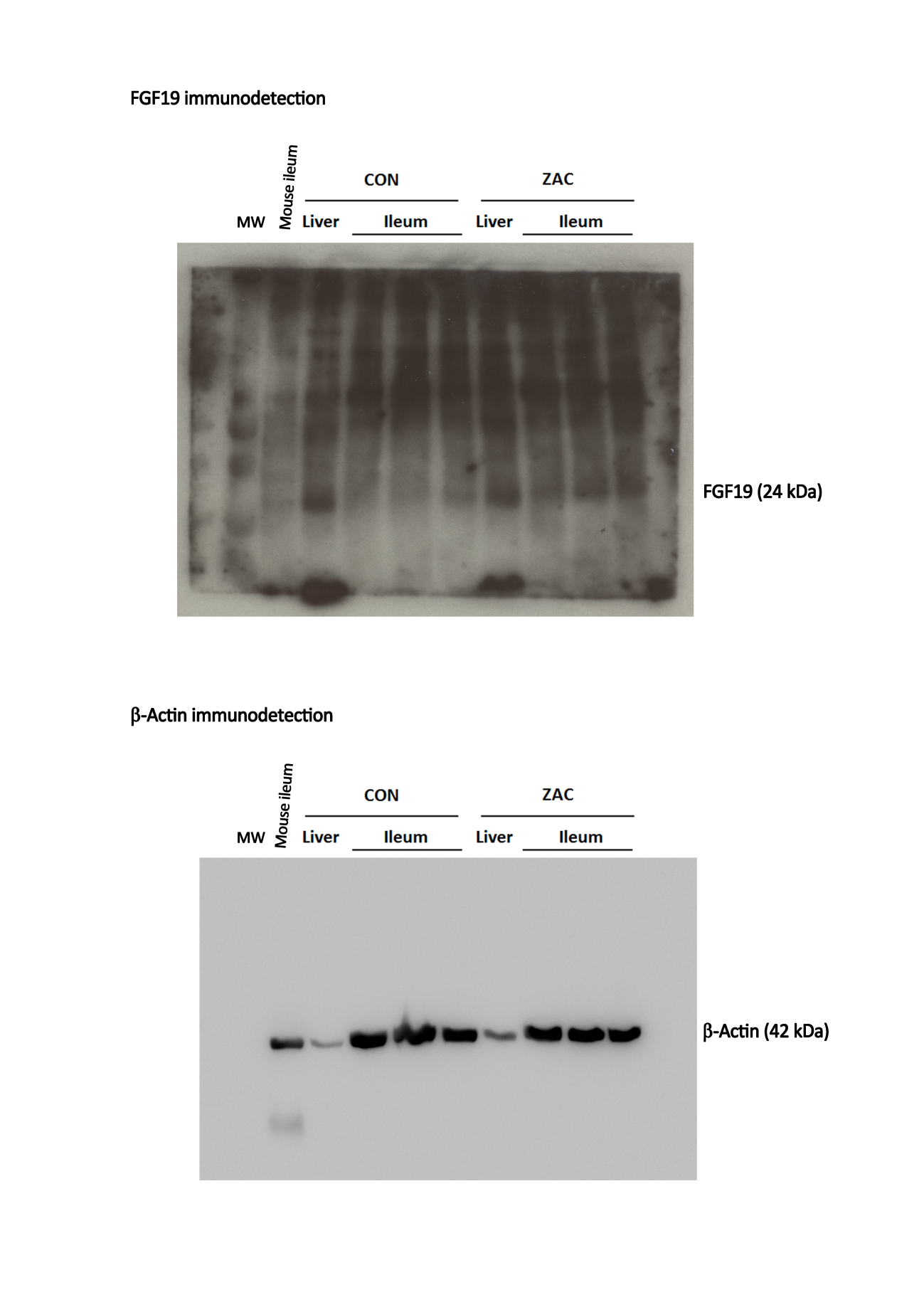

Supplement: Supplementary file 1 — Supplementary Information [file 41598_2018_32107_MOESM1_ESM.docx]
